# Transmission ways of Echinococcus granulosus in rare muscular locations of hydatid disease

**DOI:** 10.1016/j.amsu.2020.04.047

**Published:** 2020-05-22

**Authors:** Rosario VECCHIO, Veronica VECCHIO, Eva INTAGLIATA

**Affiliations:** Department of General Surgery and Medico, Surgical Specialties, University of Catania, Policlinico Vittorio Emanuele Hospital, Via S. Sofia 78, 95123, Catania, Italy

**Keywords:** Echinococcosis, Hydatid cyst, Subcutaneous tissue

## Abstract

The most common modality of transmission of the Echinococcus granulosus is through the alimentary tract. Other ways of infestation are questionable. Airborne penetration of bronchial venules to reach the heart and the systemic circulation has been advocated, but never demonstrated. Direct subcutaneous contamination through an injured skin has also been considered. Moreover, the hypothesis that a contamination different from eggs ingestion is not possible, is justified by the reason that eggs transform into larvae in the gastro-intestinal environment. Therefore, it is accepted the possibility that parasites might reach organs and tissues other than liver and lungs through a lymphatic or venous shunt that skip the portal filter. In cases of myocardial muscle or skeletal muscle involvement, it could be speculated that eggs of Echinococcus granulosus might hatch not only in the gastrointestinal tract, but also in soft tissues due to the lactic acid produced by the muscle. These unconventional ways of transmission suggest that the lifecycle of the Echinococcus is not at all known and must be revised. Issues that can help in ideating new therapies may emerge.

Dear Editor,

We read with great interest the paper by Bahjat AS et al. entitled “Hydatid cyst of the heart with mitral valve stenosis; Case report”, published in Ann Med Surg(Lond) 2019 Dec 6;49:49–52 [1]. The reported case is unique and offers the opportunity for an additional comment regarding the way of transmission of hydatid disease.

It is well recognized that the most common modality of transmission of the disease is through the alimentary tract. The adult tapeworm of Echinococcus granulosus lives in the small intestine of canids (definitive hosts) and human infestation is usually the results of ingestion of the gravid proglottids (eggs) which have been shed through feces. After ingestion, larvae are released in the small intestine and penetrate into the intestinal wall with their hooks, reaching the circulatory system and usually landing in the two filters, namely the liver and the lung.

Other ways of infestation are questionable. Airborne transmission and penetration of bronchial venules to reach the heart and the systemic circulation has been advocated [2,3], but never well demonstrated. Direct subcutaneous contamination through an injured skin has also been considered [[4], [5], [6]], but this theory of direct contact is unlikely to happen since the hands (where the contact might happen through an injury) are much less interested as a site of disease than muscular skeletal area of the body. Moreover, the hypothesis that a contamination different from eggs ingestion is not possible, is justified by the reason that eggs transform into larvae in the gastro-intestinal environment. Therefore, it is accepted the possibility that parasites might reach organs and tissues other than liver and lungs through a lymphatic or venous shunt that skip the portal filter [7,8].

In cases of myocardial muscle involvement, like the one reported by Bahjat et al. [1], or in cases were the hydatid disease is exclusively located in striated skeletal muscles as in our reported case [2], it could be speculated that eggs of Echinococcus granulosus might hatch not only in the gastrointestinal tract, but also in soft tissues due to the intervention of lactic acid produced by the underlying muscle [5,8,9]. These unconventional ways of transmission suggest that the lifecycle of the Echinococcus is not at all known and must be revised. Issues that can help in ideating new therapies may emerge. Finally, it would be interesting to know if in the case reported by Bahjat et al. [1] such hypothesis has been postulated from the anamnesis work-up of their patients.

## Provenance and peer review

Not commissioned not peer reviewed.

## Sources of funding

There are no sources of funding.

## Ethical approval

The study is exempt from ethical approval in our Hospital in Italy.

## Consent

NA.

## Author contribution

All the Authors (Vecchio R, Vecchio V, Intagliata E) contributed to conceptualization, data curation, investigation, methodology and writing. Vecchio R and Intagliata E, in addition, supervised and reviewed the manuscript.

## Registration of research studies

1. Name of the registry:
2. Unique Identifying number or registration ID:
3. Hyperlink to the registration (must be publicly accessible):

## Guarantor

Prof. Eva Intagliata.

## Declaration of competing interest

There is no conflict of interest.
